# Prediction of Proteins in Cerebrospinal Fluid and Application to Glioma Biomarker Identification

**DOI:** 10.3390/molecules28083617

**Published:** 2023-04-21

**Authors:** Kai He, Yan Wang, Xuping Xie, Dan Shao

**Affiliations:** 1 Key Laboratory of Symbol Computation and Knowledge Engineering of Ministry of Education, College of Computer Science and Technology, Jilin University, Changchun 130012, China; hekai20@mails.jlu.edu.cn (K.H.); xiexp21@mails.jlu.edu.cn (X.X.); 2 School of Artificial Intelligence, Jilin University, Changchun 130012, China; 3 College of Computer Science and Technology, Changchun University, Changchun 130022, China; shaodan@ccu.edu.cn

**Keywords:** cerebrospinal fluid, glioma biomarker, deep neural network, 68T07, 92B20

## Abstract

Cerebrospinal fluid (CSF) proteins are very important because they can serve as biomarkers for central nervous system diseases. Although many CSF proteins have been identified with wet experiments, the identification of CSF proteins is still a challenge. In this paper, we propose a novel method to predict proteins in CSF based on protein features. A two-stage feature-selection method is employed to remove irrelevant features and redundant features. The deep neural network and bagging method are used to construct the model for the prediction of CSF proteins. The experiment results on the independent testing dataset demonstrate that our method performs better than other methods in the prediction of CSF proteins. Furthermore, our method is also applied to the identification of glioma biomarkers. A differentially expressed gene analysis is performed on the glioma data. After combining the analysis results with the prediction results of our model, the biomarkers of glioma are identified successfully.

## 1. Introduction

The cerebrospinal fluid (CSF) is a body fluid that surrounds the brain and spinal cord, which makes it a perfect body fluid to reflect the pathophysiological changes in the brain [1]. Central nervous system (CNS) diseases usually are hard to detect and invasive, and biomarkers in body fluids can overcome these issues. Compared with traditional body fluids such as plasma, urine, and saliva, CSF biomarkers are more accurate for the early diagnosis of CNS diseases because of their natural advantages. Recently, many CSF biomarkers have been identified for diagnostic or therapeutic purposes of various CNS diseases, including Alzheimer’s disease, Parkinson’s disease, iron deficiency anemia, and glioma cancers [2,3,4,5,6]. CSF proteins are very important because they are promising biomarkers for CNS diseases. Although the biomarkers of certain CNS diseases have reached the clinical stage, more efficient biomarkers still need to be studied [7]. The detection of CSF proteins is still a challenge due to the high cost of biological experiments. Therefore, the prediction of CSF proteins plays a very important role in the identification of biomarkers in CSF.

Many computational methods have been proposed to predict proteins in body fluids [8,9,10,11,12,13,14,15,16]. Among these computational methods, the most successful one is based on the support vector machine (SVM) [8]. The SVM-based method was originally proposed to predict the proteins in plasma and later applied to the detection of proteins in other body fluids, including urine and saliva [9,12,13]. Although SVM-based methods have been successfully applied in multiple body fluids, this method is based on a manual negative dataset, which causes the disadvantage of limited prediction performance. Subsequently, the ranking-based computational method was proposed to overcome this issue. The ranking-based method transforms the protein-classification problem into a ranking problem [11]. This method uses a positive dataset and a background dataset to sort the data in the background set and selects proteins with a higher probability from the background set. The disadvantage of this method is that it can only sort proteins but not classify them directly. Another computational method for proteins in body fluids is based on the protein–protein interaction network [10]. Instead of directly classifying proteins in body fluids, this method aims to rank multiple body fluids and select the most likely body fluid for each protein. Similar to the ranking-based method, this method cannot directly predict the protein for a specific body fluid. Another effective method is based on deep neural networks (DNN) [14]. Compared with other methods, the DNN-based methods can usually learn more complex features to increase the representative ability and obtain a better performance. However, DNN-based methods always require a large amount of data. The performance of DNN-based methods may suffer from overfitting because the proteins in human body fluids are limited. Therefore, to obtain a more accurate method and improve the application to disease biomarker identification, an effective approach urgently needs to be presented. Although many computational methods have been proposed, these methods mainly focus on traditional body fluids, such as plasma, urine, and saliva. On the other hand, more and more CSF proteins have been identified using wet experiments. Due to the specificity of CSF for CNS diseases, the prediction method for CSF proteins needs to be studied urgently. Positive-unlabeled (PU) learning is a machine learning method that aims to perform binary classification with a small number of positive samples and a large number of unlabeled samples [17]. PU learning has been applied to many domains, including drug–target interaction and prediction of pupylation sites, and achieved some success [18,19,20,21].

In this paper, we propose a novel method based on the DNN and bagging method to predict CSF proteins based on protein features. Furthermore, we also apply this method to glioma biomarker identification. In the CSF protein prediction, four kinds of features are collected to represent each protein, and a two-stage feature-selection method is used to select the most important features. The DNN and bagging are adopted to build a computational method based on the selected protein features. This method is trained on a training dataset of CSF proteins, and the benchmarks in the independent dataset demonstrate that our method can predict CSF proteins with a relatively accurate probability. In addition, we also apply our novel method to the identification of glioma biomarkers. The rank-sum test and fold-change method are used to identify differentially expressed genes, and our novel method is used to predict potential CSF proteins. The combined results for differential genes and potential CSF biomarkers of glioma suggest that the biomarkers are successfully identified.

## 2. Results

### 2.1. Result of the Two-Stage Feature Selection

For the better prediction of CSF proteins, the two-stage feature-selection method was used to select the most important features from the protein feature vectors with 1610 dimensions. In the first stage, the *p*-value for each dimension of the features was computed based on the rank-sum test. After that, the FDR was used to calculate the q-value for each dimension based on the *p*-value. A q-value = 0.05 was used as the cutoff to remove the irrelevant features. As a result, the features of 354 dimensions were removed. In the second stage, the RFE method was applied to remove the redundant features after the previous stage. The prediction of CSF proteins was used as the base model to perform the RFE-based feature selection, and the q-value of each dimension was used as the feature importance in RFE. At each iteration in the second stage, the prediction model was trained based on some features and evaluated on the validation dataset. In this stage, the features of 1256 dimensions were used to remove the redundant features, and the features of 20 dimensions were removed at each iteration.

**Figure 1:**
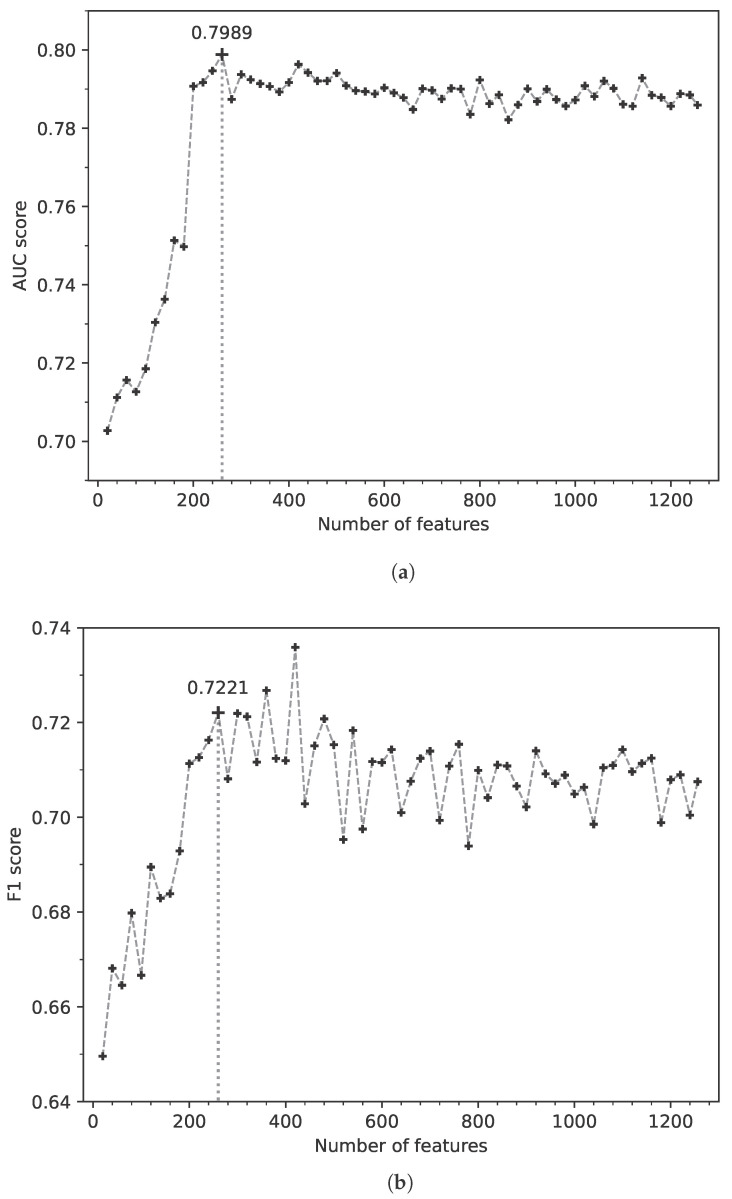
The performances on the validation dataset with different numbers of features in the second stage of the feature-selection method. (**a**) The AUC scores on the validation dataset of CSF proteins. (**b**) The F1 scores on the validation dataset of CSF proteins.

Figure 1 shows the performances of the computational method on the validation dataset at each iteration of the second stage. As is shown in this figure, both the F1 and AUC scores increased with the number of features and became stable when the number of features exceeded 260. This demonstrates that these features of 260 dimensions keep the most important information of the CSF proteins, and the other features retain a small amount of extra information. Therefore, these features of 260 dimensions were the final features for the protein classification. Finally, our two-stage feature-selection method successfully removed the irrelevant or redundant features and selected 260 protein features of 260 dimensions for the prediction of CSF proteins.

### 2.2. Comparison with other Prediction Methods

The implementation of the method proposed in this paper was based on the Python packages PyTorch, and Scikit-Learn [22,23]. Firstly, 16 sub-datasets were generated from the CSF protein dataset, and each of them contained 747 positive samples and 747 negative samples. Then, these 16 sub-datasets were used to train 16 DNN models, respectively. These DNN models had the same network architecture, and they were trained with the same hyperparameters. The input layer of each DNN had 260 units that corresponded to the number of selected features. Each DNN contained three hidden layers, and each hidden layer contained 128 neurons. A dropout probability of 0.1 was used at each hidden layer. The batch size used for each DNN was 32. The loss of each DNN for the protein classification was optimized using the Adam optimizer with a learning rate of 0.001, and each DNN was trained for 20 epochs [24]. Finally, these DNNs together composed our prediction method, and the prediction of our method was made by averaging their prediction probabilities.

The SVM, decision tree (DT), and DNN were trained to compare their performances with the proposed method [8,14]. All these methods were trained on the training dataset based on the selected features of 260 dimensions, and the hyperparameters of these methods were tuned based on the performances on the validation dataset. The performances of these methods on the independent testing dataset were reported as their benchmarks and used to compare with our method.

Table 1 shows the comparative benchmarks of these methods on the independent testing dataset. As shown in this table, our novel method reaches 0.7260, 0.7229, 0.7330, 0.7279, 0.4521, and 0.8041 in ACC, PR, RE, F1, MCC, and AUC, respectively. This table also shows that our novel method achieves much better performances than other prediction methods. In particular, our method performs much better than the other methods in the F1 and MCC metrics. Compared with DNN, our method improves by 16.09% and 5.68% in F1 and AUC. This is because our novel method can balance the positive and negative samples well. The comparative benchmarks on the independent testing dataset demonstrate that our method can predict the CSF proteins more accurately than other methods.

### 2.3. Application to Glioma Biomarker Identification

**Figure 2:**
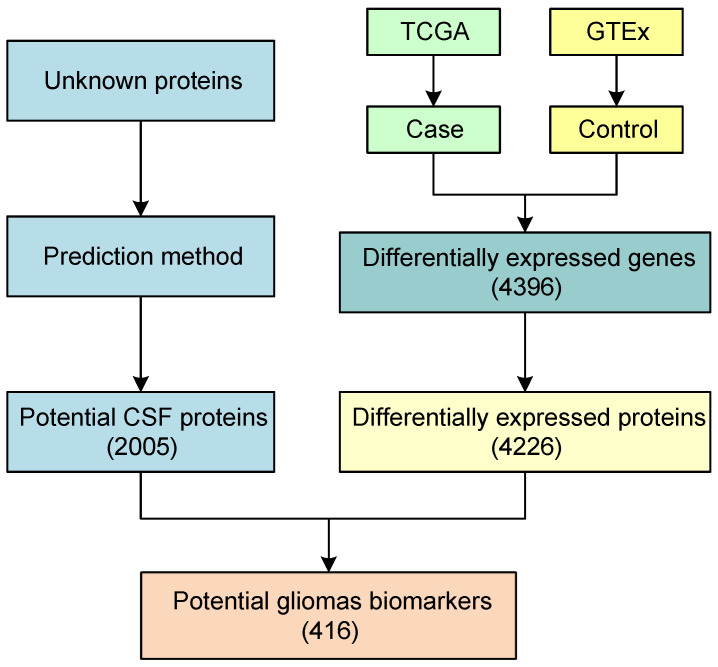
The flowchart of glioma biomarker identification.

As is shown in Figure 2, the identification of glioma biomarkers consists of two parts, including the discovery of potential CSF proteins and the identification of differentially expressed proteins in gliomas. To discover the potential CSF proteins, the prediction method was retrained on the whole dataset and then used to predict the CSF probability of unknown proteins. The probability of a CSF protein was calculated by averaging the probabilities of the DNNs for which the training datasets do not contain this protein. If the probability of an unknown protein is more than 0.5, this protein was considered a potential CSF protein. Finally, our method discovered 2005 potential CSF proteins that have not been reported before. The details of the potential CSF proteins can be found in Supplementary Table S1.

**Figure 3:**
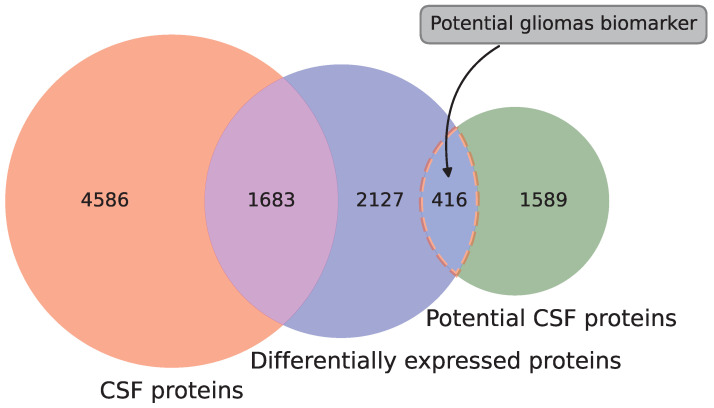
The flowchart of glioma biomarker identification.

**Figure 4:**
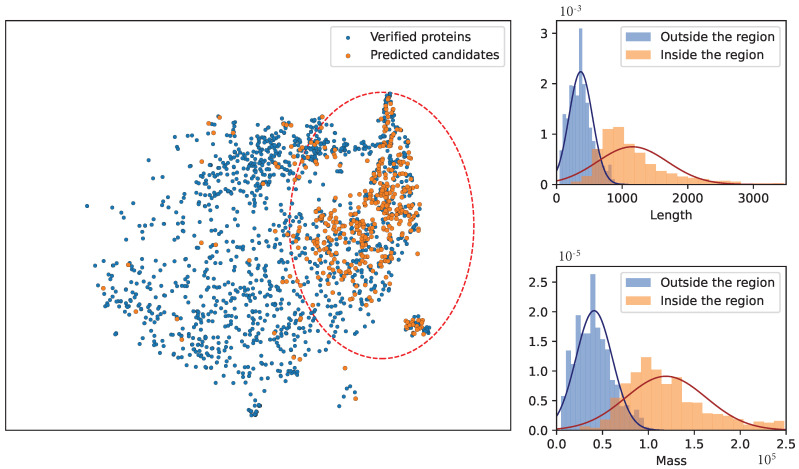
T-SNE visualization of verified proteins and predicted candidates using 260 features.

To identify the differentially expressed genes in gliomas, q-value = 0.05 and fc = 2 were used as the cutoff values of the rank-sum test and fold change method, respectively. The glioma data from the TCGA database were used as the case group, and the normal data from the GTEx database were used as the control group. As a result, 4396 differentially expressed genes (2170 up-regulated genes and 2226 down-regulated genes) were identified. After the differentially expressed genes were found, they were mapped to the proteins in CSF. These differentially expressed genes encoded a total of 4226 human proteins (2175 up-regulated proteins and 2051 down-regulated proteins). Our novel method was used to predict the CSF proteins, and the proteins that were both differentially expressed and predicted CSF proteins were considered the potential glioma biomarkers. Figure 3 shows the Venn diagram of the potential CSF proteins and differentially expressed proteins of gliomas. As is shown in this figure, among the 4226 differentially expressed proteins for gliomas, 1683 proteins were verified as CSF proteins in wet experiments and 416 proteins were predicted as CSF proteins using our novel method. Finally, 416 potential CSF biomarkers were identified using our method, and they were not reported to be secreted in CSF before. Among these potential biomarkers for gliomas, 207 were up-regulated and 209 were down-regulated. To compare the verified proteins and the predicted candidates, the t-SNE method was used to visualize these two groups of proteins using 260 features [25]. Figure 4 shows the distribution of the verified proteins and the predicted candidates. As is shown in this figure, the predicted candidates are covered by the verified proteins. This demonstrates that the predicted candidates are very close to the verified proteins. In addition, most of the predicted proteins are distributed in the upper right region. The proteins in this region are long and heavy, while the proteins in other regions are relatively short and light. This is related to the difficulty of mass spectrometry techniques for long and heavy proteins. With the development of mass spectrometry for large proteins, the predicted proteins will provide valuable references for further experimental verification [26]. Table 2 reports the potential CSF biomarkers with a predictive probability of more than 90%. The details of all these biomarkers for gliomas can be found in Supplementary Table S2.

## 3. Materials and Methods

### 3.1. Data Collection

We collected two types of data, including CSF protein data and glioma cancer data. The CSF protein data contain the protein data in CSF that will be used to train our computational method. The glioma cancer data contain the gene expression data for glioma cancers, which will be used to identify the glioma biomarkers.

#### 3.1.1. The CSF Protein Data

The CSF protein data are collected from the HBFP (Human Body Fluid Proteome) database, which is a publicly available database that has collected 11,827 experimentally validated secreted proteins for human body fluids [27]. From this database, 6269 CSF proteins that have been verified in wet experiments are retrieved for further processing. Among these proteins, 5376 proteins that have been studied by more than one study are used as the positive dataset. The remaining proteins that have not been verified by any experiments are used as the unlabeled dataset. After that, the positive dataset and unlabeled dataset are merged to form the CSF protein dataset. The dataset is then randomly divided into training, validation, and testing datasets. In detail, the training dataset contains 3226 positive samples and 11954 unlabeled samples for the training of CSF protein-prediction methods. The validation dataset contains 1075 positive samples and 1075 unlabeled samples for the search for hyperparameters. The testing dataset also contains 1075 positive samples and 1075 unlabeled samples for the evaluation of computational methods.

#### 3.1.2. The Glioma Gene Expression Data

The fragments per kilobase of transcript per million mapped reads (FPKM) data of the gliomas are downloaded from the UCSC Xena browser, including GTEx (the Genotype-Tissue Expression project) data and TCGA (The Cancer Genome Atlas) data [28]. Furthermore, 500 glioma tissues are obtained as the tumor group from the TCGA Lower Grade Gliomas (LGG) dataset. From the GTEx database [29], 443 normal tissues are collected as the control group, including the cortex, frontal cortex, anterior cingulate cortex, hippocampus, and amygdala. Then, the RNA-Seq data are merged and transformed with log2(x+1). After that, the R package “limma” is used to normalize the gene expression data of the gliomas [30].

### 3.2. Prediction of Proteins in CSF

**Figure 5:**
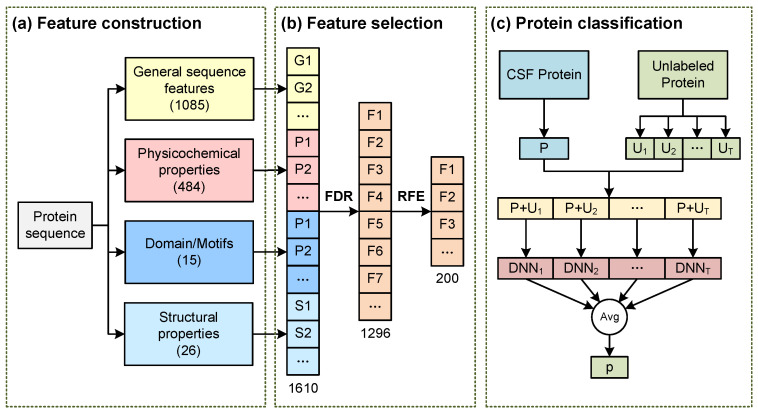
The framework to predict proteins in CSF. (**a**) Feature construction collects four groups of features for each protein; (**b**) feature selection selects the important and effective features for the protein classification; (**c**) protein classification models and predicts the proteins in CSF based on DNN and bagging methods.

The prediction of proteins in CSF is a special case of protein classification where the goal is to predict whether a protein could be secreted into the CSF. Here, we propose a prediction method to predict CSF proteins from protein features. This method is based on the DNN and bagging method. As shown in Figure 5, this computational method consists of three parts: feature construction, feature selection, and protein classification.

#### 3.2.1. Feature Construction

Feature construction collects four types of features for each protein, including general sequence features, physicochemical properties, domain/motif properties, and structural properties. The general sequence features contain 11 features directly calculated based on the amino acid sequences, such as the amino acid composition and dipeptide composition. The physicochemical properties include 24 features related to the physical or chemical properties of proteins, such as hydrophobicity and polarity. The domain/motif properties contain 11 properties related to transmembrane or motif, such as transmembrane domains and signal peptides. The structural properties include 6 features based on the protein structure, such as the secondary structure and unfoldability. All these features are computed based on the amino acid sequences with computational tools and websites [31,32]. As shown in Table 3, a total of 52 features is collected and represented as a vector of length 1610. For each dimension of these protein features, the empty values are filled with the median of the corresponding feature vectors. Then, the protein features are standardized by subtracting the mean and dividing by the standard deviation.

#### 3.2.2. Feature Selection

The feature construction has collected many protein features that could contain irrelevant features and redundant features. Irrelevant features and redundant features usually cannot improve the performance of the computational method but will affect the generalization performance. Therefore, irrelevant features and redundant features need to be removed for better prediction of CSF proteins. A two-stage feature-selection method is adopted here to remove the irrelevant features and redundant features. This feature-selection method is based on the rank-sum test, false discovery rate (FDR), and recursive feature elimination (RFE) [14,33]. In the first stage, the rank-sum test and FDR are used to remove the irrelevant features. For each dimension of the feature vectors, the *p*-value is computed based on the CSF label with the rank-sum test. Then, the q-value is calculated based on the *p*-value and FDR method. If the *p*-value is more than the q-value, this dimension would be considered an irrelevant feature. After comparing all the *p*-values and q-values, the irrelevant features would be removed from these selected protein features. In the second stage, the RFE method is used to remove redundant features from the result of the first stage. The *p*-values calculated in the first stage are used as the feature importance in the RFE method. At this stage, a small number of features is removed at each iteration recursively. At each iteration, the features with the least feature importance are removed and the classifier are retrained and evaluated. After the features are removed, the optimal feature subset is selected based on the performance on the validation dataset. These features are the final features selected using the two-stage feature-selection method and will be used to train the protein classification model.

#### 3.2.3. Protein Classification

Protein classification aims to build the classifier for CSF proteins based on the selected protein features. As is shown in Figure 5, protein classification is based on the DNN and bagging methods [34,35]. Protein classification only requires CSF proteins and unlabeled proteins, and it is trained with the following steps. Firstly, the CSF proteins constitute the dataset P, and the unknown proteins constitute the dataset U. Secondly, dataset U is divided into T unlabeled sub-datasets of the same size {U1, U2, …, UT}. Then, T positive sub-datasets are generated from the P dataset using random sampling, and these positive sub-datasets have the same number of samples as the unlabeled sub-datasets. After that, T positive sub-datasets and T unlabeled sub-datasets are merged into T different binary classification datasets. T DNNs are trained based on these T binary classification datasets individually. The final prediction of a protein is made by averaging the probabilities of these DNNs of which the training dataset does not contain this protein.

All these DNNs have the same network architecture, and each of them is composed of one input layer, multiple hidden layers, and one output layer. The input layer corresponds to the number of features selected during feature selection. Each hidden layer has multiple neurons to transform its input into complex features [34]. Each neuron is connected with all the input features and computed with the linear transformation of these input features. The input of the first hidden layer is the selected features, and the input of the others is the output of the last hidden layer. For each neuron, its output value is computed as a weighted sum of all the input values and then processed through a non-linear activation function. These hidden layers adopt the ReLU as the nonlinear activation function, and the computation of the hidden layer is defined as follows:

(1) $$h_{i}^{l+1}=\max\left(0,w_{i}^{l+1}\cdot h^{l}+b_{i}^{l+1}\right),$$

where $h_{i}^{l+1}$ is the output value of the *i*-th neuron, $w_{i}^{l+1}$ and $b_{i}^{l+1}$ are the weight and bias, respectively, of the *i*-th neuron, and $h^{l}$ is the input of this layer. The output layer contains two neurons, representing positive and negative. The computation of the output layer is the linear transformation of the input features that are the output of the last hidden layer. The output value of *i*-th neuron in the output layer is defined as follows:

(2) $$o_{i}=h\cdot \alpha_{i}+\beta_{i},$$

where $o_{i}$ is the output value of *i*-th neuron, $\alpha_{i}$ and $\beta_{i}$ represent the weight and bias, respectively, of the *i*-th neuron, and *h* is the output value of the last hidden layer. Then, the softmax function is used to transform the output values into the predictive probability *p*, which is defined as follows:

(3) $$p=\frac{\exp o_{2}}{\exp o_{1}+\exp o_{2}}.$$

For a protein, if the predictive probability *p* is more than 0.5, this protein would be considered a CSF protein. Cross entropy is adopted as the loss function of the binary classification task, which is defined as follows:

(4) $$L=-\frac{1}{N}\sum_{i=1}^{N}\left(y_{i}\log p_{i}+\left(1-y_{i}\right)\log\left(1-p_{i}\right)\right),$$

where $y_{i}$ and $p_{i}$ are the CSF label and probability, respectively, of the *i*-th protein and *N* is the number of proteins.

### 3.3. Identification of Differentially Expressed Genes

The rank-sum test, FDR, and fold change are applied to identify differentially expressed genes in gliomas. Firstly, the rank-sum test is used to calculate the *p*-value for each gene. Then, the FDR is employed to estimate the statistical significance based on the *p*-value and calculate the q-value. The fold change is adopted to measure the difference between each gene in cancer and normal tissue, and it is defined as follows:

(5) $$FC_{i}=\frac{\bar{c_{i}}}{\bar{n_{i}}}=\frac{\sum c_{ij}}{\sum n_{ij}},$$

where $c_{ij}$ is the expression value of gene *i* in cancer tissues from patient *j* and $n_{ij}$ is th expression value of gene *i* in normal tissue from patient *j*.

These two measures are often used to identify differentially expressed genes. The cutoff of the q-value used in this work is 0.05. The genes with FC more than 2 are considered over-expressed genes, while those with FC less than 0.5 are considered under-expressed genes.

### 3.4. Evaluation

To evaluate the performances of the CSF protein-prediction model, the accuracy (ACC), precision (PR), recall (RE), F1 score (F1), Matthew’s correlation coefficient (MCC), and Area under the ROC Curve (AUC) are employed. Higher values demonstrate better classification performances for all these metrics. The ACC, PR, RE, F1, and MCC metrics are defined as follows:

(6) $$\mathrm{ACC}=\frac{TP+TN}{TP+TN+FP+FN},$$

(7) $$\mathrm{PR}=\frac{TP}{TP+FP},$$

(8) $$\mathrm{RE}=\frac{TP}{TP+FN},$$

(9) $$F1=\frac{2TP}{2TP+FP+FN},$$

(10) $$\mathrm{MCC}=\frac{TP\times TN-FN\times FP}{\sqrt{\left(TP+FN)\times (TP+FP)\times (TN+FP)\times (TN+FN\right)}},$$

where *TP*, *TN*, *FP*, and *FN* represent the number of true positives, true negatives, false positives, and false negatives, respectively.

## 4. Discussion

In this work, a computational method is proposed to predict the proteins in CSF based on protein features. A two-stage feature selection is employed to select the most important features for the prediction. To detect the proteins in CSF, DNN and bagging are adopted to build classifiers for the prediction of proteins in CSF. Our novel method also can be used to detect proteins in CSF from unknown proteins. Compared with the SVM-based method, our method does not need the generation of a manual negative dataset. This would improve the prediction performances of potential CSF proteins because no artificial knowledge is introduced. Although the ranking-based method and our method do not require a manual negative dataset during construction, our method is still superior to the ranking-based method. This is because the ranking-based method can only sort unknown proteins, but our method can predict whether a protein is secreted into the CSF. In terms of evaluation performances, our method is much better than other methods mainly in the F1 and MCC indicators. This demonstrates that our method balances positive and negative samples well. These improvements also affirm the effectiveness of our method. The bagging-based strategy can not only improve the prediction effect but also balance the positive and negative samples well. All these advantages would improve the accuracy of the potential CSF proteins predicted by our method and the application of the prediction results. Although our computational method has achieved a good performance, there are still some efforts that can be made to improve the prediction of CSF proteins. The protein features collected in this study are limited, and more features could be collected in the future to increase the expressive ability of the computational method. In addition, more advanced feature-selection methods should also be considered to improve the performance.

The potential CSF proteins are also applied to identify biomarkers for gliomas. First, the rank-sum test and fold change method were adopted to identify the differentially expressed genes. After that, the analysis results were combined with the potential CSF proteins to identify the biomarkers of gliomas, and the glioma biomarkers were found successfully. The application of our prediction method to glioma biomarker identification provides a new idea for the identification of biomarkers in CNS diseases. There are too many glioma biomarkers obtained in this study, and they have not been verified in any experiments. To improve the early diagnosis of CNS diseases, the biomarkers identified in our study still need to be verified with experiments in the future. Furthermore, the biomarkers can also be combined with machine learning methods to build early diagnosis models for CNS diseases [36,37].

## 5. Conclusions

In this study, we propose a novel method to predict CSF proteins and apply it to identify glioma biomarkers. In this prediction method, feature vectors of dimension 1610 are constructed, and 260 features are selected from them based on a two-stage feature-selection method. After that, DNN and bagging are used to model CSF proteins based on these selected features. Furthermore, our computational method is also used to predict potential CSF proteins. The differentially expressed genes are identified with the rank-sum test and fold change method, and correspondingly, 4226 differentially expressed proteins are identified. By fusing these two experimental results, 416 proteins are found to be differentially expressed and predicted to be secreted into the CSF.

In the future, we would like to further improve the performance of the prediction method through a more effective network architecture and apply the prediction method to find biomarkers for other CNS diseases.

## Figures and Tables

**Table 1:** The comparative benchmarks on the independent testing datasets of CSF.

Methods	ACC	PR	RE	F1	MCC	AUC
SVM	0.6158	**0.9003**	0.2605	0.4040	0.3293	0.7891
DT	0.6140	0.6923	0.4102	0.5152	0.2496	0.6140
DNN	0.6726	0.8367	0.4288	0.5670	0.3953	0.7697
**Our method**	**0.7260**	0.7229	**0.7330**	**0.7279**	**0.4521**	**0.8041**

The best results are in bold.

**Table 2:** The potential glioma biomarkers identified using our method.

Id	Accession	FC	Probability	q-Value	Type
1	Q9H4X1	3.19	92.66%	0.0218	up
2	P28370	3.18	92.15%	0.0007	up
3	P49368	2.38	93.18%	0.0073	up
4	O14497	2.29	90.60%	0.0094	up
5	Q9P2E5	2.21	90.93%	0.0065	up
6	Q9UPP2	0.14	90.21%	0.0105	down
7	Q12955	0.14	96.78%	0.0053	down
8	Q14643	0.24	91.32%	0.0207	down
9	O15020	0.32	91.48%	0.0224	down
10	Q70CQ2	0.36	95.00%	0.0040	down
11	Q96M86	0.47	93.95%	0.0109	down

**Table 3:** The four types of features collected for the prediction of CSF proteins.

Type	Feature Name	Length
General sequence properties	Sequence length	1
	Mass	1
	Amino acid composition	20
	Dipeptides composition	400
	Normalized Moreau–Broto autocorrelation descriptors	90
	Moran autocorrelation	90
	Geary autocorrelation descriptors	90
	Quasi-sequence-order descriptors	160
	Pseudo-amino acid composition	150
	Amphiphilic pseudo-amino acid composition	80
	Total amino acid property	3
Physicochemical properties	Hydrophobicity	21
	Normalized Van der Waals volumes	21
	Polarity	21
	Polarizability	21
	Charge	21
	Solvent accessibility	21
	Surface tension	21
	Molecular weight	21
	Solubility in water	21
	No. of hydrogen bond donors in side chain	21
	No. of hydrogen bond acceptors in side chain	21
	CLogP	21
	Amino acid flexibility index	21
	Protein–protein Interface hotspot propensity—Bogan	21
	Protein–protein Interface (PPI) propensity—Ma	21
	Protein–DNA Interface propensity—Schneider	21
	Protein–DNA Interface propensity—Ahmad	21
	Protein–RNA Interface propensity—Kim	21
	Protein–RNA Interface propensity—Ellis	21
	Protein–RNA Interface propensity—Phipps	21
	Protein–ligand binding site propensity—Khazanov	21
	Protein–ligand valid binding site propen—Khazanov	21
	Propensity for protein–ligand polar and atom–Imai	21
	Isoelectric point	1
Domains/motifs properties	Twin-arginine signal peptide	1
	Transmembrane domains	1
	Signal peptide	1
	Number of glycosylation sites	1
	Glycosylation presence	1
	Phosphorylation sites	1
	Cleavage site	3
	Subcellular location	3
	Percentage of coil content	1
	Number of predicted motif sites	1
	Transmembrane helices	1
Structural properties	Secondary structure	21
	Unfoldability	1
	Fldbin charge	1
	Number of disordered regions	1
	Longest disordered regions	1
	Number of disordered residues	1

## Data Availability

Not applicable.

## Supplementary Materials

The following supporting information can be downloaded at: https://www.mdpi.com/article/10.3390/molecules28083617/s1.

## Abbreviations

The following abbreviations are used in this manuscript:

CSF	Cerebrospinal fluid
CNS	Central nervous system
SVM	Support vector machine
DNN	Deep neural network
PU	Positive unlabeled
FDR	False discovery rate
RFE	Recursive feature elimination
FC	Fold change
MCC	Matthew’s correlation coefficient
AUC	Area under the ROC Curve
